# Cannabinoid Receptor Type 2 Agonist, GW405833, Reduced the Impacts of MDA‐MB‐231 Breast Cancer Cells on Bone Cells

**DOI:** 10.1002/cam4.70709

**Published:** 2025-02-20

**Authors:** Ingon Inson, Chartinun Chutoe, Janjira Kanjanapipak, Kornkamon Lertsuwan

**Affiliations:** ^1^ Department of Biochemistry, Faculty of Science Mahidol University Bangkok Thailand; ^2^ Center of Calcium and Bone Research (COCAB), Faculty of Science Mahidol University Bangkok Thailand

**Keywords:** bone interaction, breast cancer, CB2 agonist, metastasis, osteoblast, osteoclast

## Abstract

**Aim:**

Breast cancer frequently metastasizes to bones. The interaction between breast cancer cells and bone cells results in osteolytic lesions by disrupting the balance between osteoblast‐mediated bone production and osteoclast‐mediated bone resorption. This study aims to investigate the effects of the cannabinoid receptor type 2 (CB2) agonist, GW405833, on interactions between breast cancer cells and osteoblasts as well as its impact on breast cancer‐induced osteoclastogenesis.

**Materials & Methods:**

MDA‐MB‐231, UMR‐106, RAW 264.7 cells were used to represent breast cancer cells, osteoblast‐like cells and macrophage‐osteoclast precursor cells, respectively. Cell viability was evaluated by MTT assay, and breast cancer cell invasion was assessed by Transwell invasion assay. Tartrate‐resistant acid phosphatase (TRAP) staining was utilized to evaluate osteoclastogenesis.

**Results:**

Our results demonstrated that GW405833 disrupted MDA‐MB‐231‐induced UMR‐106 cell death and promoted UMR‐106 cell viability. The underlying mechanism of these effects was determined in this study. GW405833 reduced AKT phosphorylation in MDA‐MB‐231 cells without affecting mTOR protein expression or its phosphorylation. Conversely, in UMR‐106 cells, GW405833 induced AKT and mTOR phosphorylated protein. Furthermore, the mTOR inhibitor reversed the GW405833‐induced recovery of UMR‐106 cell viability under MDA‐MB‐231‐derived conditioned media (CM) exposure. These findings underscore the critical role of the AKT/mTOR pathway in mediating GW405833's inhibitory effects on cancer‐bone interactions. Additionally, GW405833 suppressed osteoblast‐enhanced breast cancer cell invasion and the expression of invasion‐related proteins in both cell types, along with reducing osteoclastogenic factors induced by MDA‐MB‐231 CM in UMR‐106 cells and suppressing MDA‐MB‐231 CM‐enhanced osteoclastogenesis in RAW 264.7 cells.

**Conclusion:**

This study highlights the therapeutic potential of cannabinoid receptor agonist for treating breast cancer bone metastasis and bone‐related complications.

## 1 Introduction

More than 60% of metastatic breast cancer is bone metastasis, leading to a drastic reduction in patients' survival rate with increased skeleton‐related events, including bone fractures, bone pain, and hypocalcemia [1]. In addition, bone metastasis was shown to correlate with and could be the origin for multiple organ relapses later. Therefore, understanding the mechanisms behind breast cancer bone metastasis is highly valuable for therapeutic application [2]. Breast cancer bone metastasis was shown to cause osteolytic lesions, where higher bone degradation and lower bone formation were observed. As a major metastatic organ for breast cancer, the interaction between breast cancer and the bone microenvironment was shown to regulate and, in part, facilitate breast cancer bone metastasis. Previous studies showed that initial breast cancer bone metastasis occurs via the interaction between soluble cytokines released from the bone microenvironment, especially chemokine (C‐X‐C motif) ligand 12 (CXCL12), and its receptor, C‐X‐C chemokine receptor type 4 (CXCR4) on breast cancer cells, leading to the attraction of breast cancer to bone [3, 4, 5]. Once arrived in bone, cancer cells secreted several cytokines, such as parathyroid hormone‐related protein (PTHrP), interleukin‐6 (IL‐6) and tumor necrosis factor (TNF), which could serve as osteoclastogenic factors to promote osteoclast differentiation and the bone degradation process. At the same time, the degraded bone matrix released trapped growth factors, including transforming growth factor‐beta (TGF‐beta), insulin‐like growth factor (IGF), platelet‐derived growth factor (PDGF) and bone morphogenetic proteins (BMPs). All of which could, in turn, facilitate breast cancer growth (as reviewed in [2, 6, 7]). While the interaction between osteoclasts and breast cancer has been elucidated extensively, little is known about the interaction between breast cancer and osteoblasts during cancer bone metastasis. Previous studies showed that breast cancer cell colonization occurred at the osteogenic or osteoblast‐rich area in bone, and the number of osteoblasts reduced as the cancer growth progressed [8, 9]. This phenomenon suggested that osteoblasts may also play an important role in breast cancer bone colonization. Previous studies showed that osteoblast‐derived conditioned medium (CM) in a co‐culture model was shown to protect breast cancer cell viability under stress induction [8]. On the other hand, CM from metastatic/osteolytic breast cancer cells, MDA‐MB‐231, was shown to suppress osteoblast survival and differentiation, resulting in lower bone formation [10, 11, 12]. Breast cancer CM also induced inflammatory cytokine production from osteoblasts, which could further support breast cancer cell colonization and osteoclast differentiation, leading to higher bone degradation during breast cancer metastasis [13, 14]. However, how other cytokines in the bone microenvironment affect this interaction is not known.

Several signaling molecules and pathways in the bone microenvironment were reported to regulate bone homeostasis, including the endocannabinoid system (ECS). The ECS consists of two primary receptors (cannabinoid receptor 1 (CB1) and cannabinoid receptor 2 (CB2)), endocannabinoids, and the requisite enzymes for their synthesis and degradation [15]. While the CB1 receptor is predominantly found in the central nervous system, the CB2 receptor is primarily distributed in peripheral tissues, including bone [16]. Previous studies have demonstrated that the deficiency of CB2 receptors leads to increased osteoclast numbers, higher bone turnover, reduced osteoblast precursors, and diminished bone density [17]. Furthermore, CB2 receptor agonists have been shown to decrease RANKL expression, consequently reducing osteoclast formation and function, thereby ameliorating osteoporosis in preclinical studies [18]. Additionally, endocannabinoid ligands such as 2‐AG and AEA are produced by various types of cells in the bone microenvironment, including osteoclasts, osteoblasts, and osteocytes [19]. All of these suggest a role of the ECS in maintaining bone function and homeostasis. Given its potential involvement in bone homeostasis regulation, this system could be another group of cytokines encountered by breast cancer cells during bone metastasis. The CB2 receptor is commonly expressed in many cell types, including breast cancer, osteoblast, and osteoclast precursor cells [19, 20, 21, 22, 23]. Previous studies have demonstrated the relationship between CB2 receptor function and the inhibition of CXCR4, CXCL12, and MMP9 in various study models [24, 25, 26, 27]. Given that the CXCR4/CXCL12/MMP9 axis plays a critical role in breast cancer bone metastasis (as reviewed in [28]), CB2 activation may influence breast cancer bone metastatic potential. However, the precise role of the ECS in the interplay between cancer and bone cells during breast cancer metastasis remains unclear. Our previous research has revealed that breast cancer cells secreted factors that induced osteoblast cell death, and this interaction was suppressed by the presence of a CB2 agonist [12]. Nevertheless, the specific factors and signaling pathways governing this interaction, as well as the effects of CB2 agonist exposure on cancer and bone cell interactions, remain poorly understood. Therefore, this study aimed to elucidate the effects and underlying mechanisms of CB2 receptor agonist on breast cancer and osteoblast interaction. Specifically, we investigated the effects of the CB2 receptor agonist on the survival and metastatic potential of breast cancer cells under cancer‐bone interaction, as well as its impact on osteoclast development under the influence of breast cancer. Altogether, these findings provided valuable information about the potential role and therapeutic implications of CB2 agonist in impeding breast cancer bone metastasis.

## 2 Materials and Methods

### 2.1 Cell Culture and Chemicals

The cell lines used in this study included UMR‐106 (RRID: CVCL_3617; ATCC: CRL‐1661), RAW 264.7 (RRID: CVCL_0493; ATCC: TIB‐71), and MDA‐MB‐231 (RRID: CVCL_0062; ATCC: HTB‐26), representing osteoblast‐like cells, mouse macrophage precursor cells, and breast cancer cells, respectively. These cell lines were acquired from the American Type Culture Collection (ATCC, VA, USA). To ensure optimal growth conditions, the cells were cultured in a humidified incubator at a temperature of 37°C with 5% CO_2_. Dulbecco's modified Eagle's medium (DMEM) (Sigma‐Aldrich, MO, USA) supplemented with 1% (v/v) penicillin–streptomycin (Gibco, TX, USA) and 10% (v/v) fetal bovine serum (FBS) (Sigma‐Aldrich, MO, USA) was used as the culture medium. Cell sub‐culturing was performed according to the guidelines provided by the ATCC. The CB2 agonist, GW405833 (G1421) was purchased from Sigma‐Aldrich (Sigma‐Aldrich, MO, USA). Rapamycin, an mTOR inhibitor, was purchased from Cell Signaling Technology Inc. (Cell Signaling Technology, MA, USA).

### 2.2 Dose Response Study on Cell Viability

MDA‐MB‐231 and UMR‐106 cells were seeded with an initial density of 1.0 × 10^4^ cells/well in a 96‐well plate. After 24 h, cells were exposed to a various concentrations of GW405833 (CB2 agonist) from 0 to 100 μM. MDA‐MB‐231 and UMR106 were treated with GW405833 for 48 and 72 h, respectively. Thereafter, cell viability was determined by adding 0.5 mg/mL of 3‐(4,5‐dimethylthazolk‐2‐yl)‐2,5‐diphenyl tetrazolium bromide (MTT) solution (M6494, Invitrogen, CA, USA). Following 3 h incubation at 37°C, dimethyl sulfoxide (DMSO) (1,167,431,000, Millipore, MA, USA) was added to each well to dissolve the formazan crystals produced by MTT reduction. The resulting solution's absorbance was measured at a wavelength of 595 nm using a microplate reader (Multiskan EX, Thermo Fisher Scientific, MA, USA). The IC_50_ of cells in response to the CB2 agonist was determined by using non‐linear regression analysis using GraphPad Prism 9 software (GraphPad Software Inc., CA, USA).

### 2.3 Cell‐Derived Conditioned Media (CM)

CM was collected from MDA‐MB‐231 cells with an initial density of 5 × 10^5^ cells/well in a six‐well plate. After 24 h, the cells were exposed to 15 μM CB2 agonist, GW405833, or 30 nM mTOR inhibitor, rapamycin, for 48 h for the pretreatment‐CM condition. For the cotreatment‐CM condition, MDA‐MB‐231 cells were not exposed to any compound and continued culturing with media. Following 72 h of cell culturing, the cells were washed with phosphate‐buffered saline (PBS) and then incubated with serum‐free DMEM for an additional 48 h. The CM was collected and filtered through a syringe filter with a pore size of 0.22 μm (Sartorius Stedim Biotech GmbH, Göttingen, Germany) to remove debris and cells. The CM was stored at −20°C until used in the experiments. The timeline of CM preparation was described in Figure 2A.

### 2.4 Cytotoxic Effect Study of MDA‐MB‐231‐Derived CM on UMR106 Cell Viability

UMR‐106 cells were seeded on a 96‐well plate at a density of 1.0 × 10^4^ cells/well and treated with 15 μM GW405833, 50% (v/v) CM, rapamycin, or the combination of each treatment for 72 h. Cell viability assay was then utilized following the previously mentioned method. Briefly, 0.5 mg/mL MTT (Invitrogen) was added to the cell culture medium. After 3‐h of incubation at 37°C, DMSO (Millipore) was added to each well before measuring the absorbance by using a microplate reader (Multiskan EX, Thermo Fisher) at a wavelength of 595 nm.

### 2.5 Western Blot Analysis

Thirty micrograms of protein samples were loaded onto an SDS‐PAGE gel (Invitrogen, CA, USA). After transferring the proteins to a nitrocellulose blotting membrane (GE Healthcare, TX, USA), blocking was performed using a 5% (w/v) bovine serum albumin (BSA) solution (Capricorn Scientific, Hessen, Germany) for 2 h at room temperature to prevent non‐specific binding. Subsequently, the membranes were subjected to overnight incubation at 4°C with primary antibodies. Primary antibodies included β‐actin (RRID: AB_476693; #A2066) was purchased from Sigma‐Aldrich (Sigma‐Aldrich, MO, USA), MMP‐9 (RRID: AB_2144612; #2270), and mTOR (RRID: AB_2105622; #2983) were purchased from Cell Signaling Technology Inc. (Cell Signaling Technology, MA, USA). Phospho‐p44/42 MAPK (Erk1/2) (RRID: AB_331646; #9101), Phospho‐mTOR (RRID: AB_10691552; #5536), and Phospho‐Akt (Ser473) (RRID: AB_2315049; #4060) were kindly provided by Rutaiwan Tohtong and Thaned Kangsamaksin, respectively, at Mahidol University. A secondary antibody, anti‐rabbit IgG conjugated with horseradish peroxidase (RRID: AB_2099233; #7074) from Cell Signaling Technology Inc. (Cell Signaling Technology, MA, USA) was used for detection. To enhance the signal of protein expression, an enhanced chemiluminescence (ECL) substrate (Millipore, MA, USA) was employed. The protein expression was then visualized using the Azure 600 imaging system (Azure Biosystems, CA, USA) and analyzed by ImageJ software (National Institutes of Health, MD, USA).

### 2.6 Transwell Invasion Assay

For monoculture, the top insert chamber was coated with a 1:10 diluted Matrigel (#356234, Corning, NY, USA) in serum‐free culture medium. After gel congelation, MDA‐MB‐231 cells at 5 × 10^4^ cells were seeded in serum‐free culture medium with 15 μM GW405833 in the inserted upper chamber, while the bottom chamber of the transwell plate (Falcon, AZ, USA) was filled with DMEM supplemented with 20% FBS. On the other hand, the co‐culture system consisted of MDA‐MB‐231 cells in serum‐free media in the upper chamber and UMR‐106 cells at a density of 5 × 10^4^ cells in 20% FBS containing media in the bottom chamber. The cells were incubated for 12 h with or without 15 μM GW405833. After that, the transwell insert was stained with 0.5% (w/v) crystal violet (V5265, Sigma‐Aldrich, MO, USA) at room temperature, and the non‐invasive cells on the upper side of the insert were removed by swabbing. The remaining invasive cells on the underside were observed using a light microscope (Model MBL3200, A. KRÜSS Optronic GmbH, Hamburg, Germany). The number of invasive cells was quantified using ImageJ software (National Institutes of Health).

### 2.7 Quantitative Real‐Time PCR (qRT‐PCR)

Cells were treated with 15 μM GW405833 for 72 h. Then, total RNA was extracted from UMR‐106 cells using the RNeasy kit (Qiagen, Hilden, Germany) following the manufacturer's protocol to investigate OPG, RANKL, CXCL12 and Hprt1 expression. For MDA‐MB‐231 cells, 15 μM GW405833 was applied for 48 h. Then, RNA was isolated using TRIzol reagent (Invitrogen, USA) according to the manufacturer's protocol to investigate CXCR4 and β‐actin expression. Total RNA was reverse transcribed into cDNA using the iScript cDNA synthesis kit (Bio‐rad, CA, USA) and a thermal cycler (model MyCycler; Bio‐rad, CA, USA) following the manufacturer's recommendations. PCR primers were described in Table 1. qRT‐PCR was performed using the Bio‐rad MiniOpticon and iTaq Universal SYBR Green Supermix (Bio‐rad, CA, USA) and amplified using a Bio‐Rad CFX Connect qPCR machine (Bio‐rad, CA, USA).

**TABLE 1:** qPCR primers details used in this study.

Gene name		Accession no.	Primer sequence	Annealing temperature (°C)
Receptor activator of nuclear factor kB ligand (RANKL)	Forward	NM_057149	5′‐TCGCTCTGTTCCTGTACT‐3′	47.4
	Reverse		5′‐AGTGCTTCTGTGTCTTCG‐3′	
Osteoprotegerin (OPG)	Forward	NM_012870	5′‐ATTGGCTGAGTGTTCTGGT‐3′	45.2
	Reverse		5′‐CTGGTCTCTGTTTTGATGC‐3′	
Hypoxanthine phosphoribosyltransferase 1 (Hprt1)	Forward	NM_012583	5′‐GGCCAGACTTTGTTGGATTTG‐3′	52.7
	Reverse		5′‐CTTTCGCTGATGACACAAACAT‐3′	
C‐X‐C Motif Chemokine Ligand 12 (CXCL12)	Forward	NM_022177	5′‐GTCTAAGCAGCGATGGGTTC‐3′	60
	Reverse		5′‐GAATAAGAAAGCACACGCTGC‐3′	
C‐X‐C Motif Chemokine Receptor 4 (CXCR4)	Forward	NM_001348059.2	5′‐CCCAAACGCGCCAAGTGATAAA‐3′	60.4
	reverse		5′‐TGTATATCTCCTCCCCCAAGCG‐3′	
β‐Actin	Forward	NM_031144	5′‐AAACTG GAACGG TGAAGGTG‐3′	54
	Reverse		5′‐AGAGAAGTGGGGTGGCTTT‐3′	

### 2.8 Tartrate‐Resistant Acid Phosphatase (TRAP) Staining

RAW 264.7 cells were seeded at a density of 9.5 × 10^3^ cells/well in a 24‐well plate and allowed to attach overnight. Cells were primed with 50 ng/mL RANKL (462‐TEC, R&D Systems, MN, USA) for 48 h. Afterward, cells were washed with PBS. For the positive control, cells were treated with DMEM containing 50 ng/mL RANKL. For the CM‐treated condition, cells were treated with 10% (v/v) CM with or without 10 μM GW405833 for 48 h. Subsequently, cells were washed with PBS and stained with TRAP chromogenic substrates (PMC‐AK04F‐COS, Cosmo Bio, Tokyo, Japan) for 60 min at 37°C. The number of TRAP‐positive multinucleated osteoclasts containing at least three nuclei was manually counted after taking images under a light microscope (Model MBL3200, A. KRÜSS Optronic GmbH, Hamburg, Germany).

### 2.9 Statistical Analysis

Data obtained from the experiments were subjected to statistical analysis using GraphPad Prism 9 software (GraphPad Software Inc., CA, USA). To compare multiple groups, a one‐way analysis of variance (ANOVA) followed by a Tukey post‐test was performed. A *t*‐test was used to compare two groups. Statistical significance was determined by a *p*‐value of ≤ 0.05.

## 3 Results

### 3.1 GW405833 Exposure on Both Breast Cancer and Osteoblast Disrupted MDA‐MB‐231 Breast Cancer‐Induced UMR‐106 Osteoblast Cell Death while Promoted UMR‐106 Cell Viability

**FIGURE 1:**
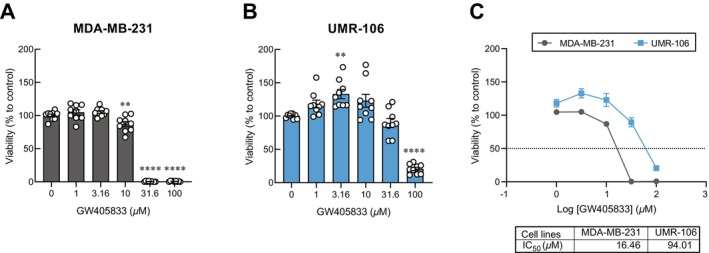
GW405833 had a greater suppressive effect on MDA‐MB‐231 cell viability as compared to UMR‐106 cells. (A) MDA‐MB‐231 cell viability and (B) UMR‐106 cell viability when treated with GW405833 at a concentration 0–100 μM. (C) Comparative cell viability between MDA‐MB‐231 cells and UMR‐106 cells when treated with 0–100 μM of GW405833 and IC_50_ values of both cells. The results were expressed as mean ± SEM of three independent biological replicates with three technical replicates each. (**p < 0.01, ****p < 0.0001).

**FIGURE 2:**
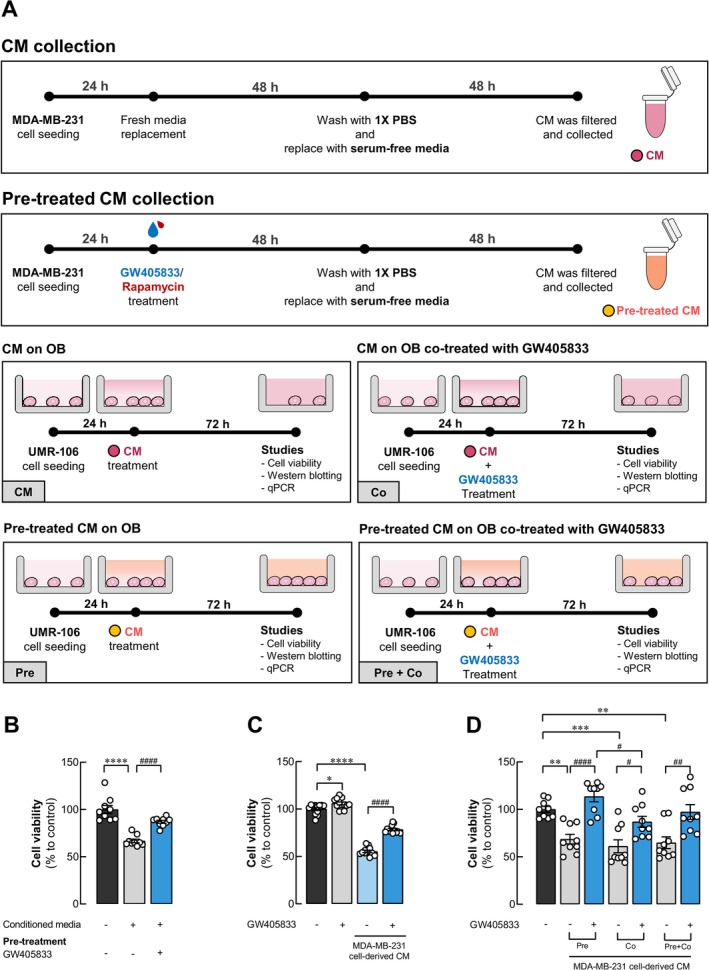
GW405833 protected UMR‐106 osteoblast cells from MDA‐MB‐231 breast cancer‐derived conditioned media. (A) Diagram showed the protocol for conditioned media (CM) preparations and timeline for each condition. Cell viability was determined by MTT assay after treatment with (B) MDA‐MB‐231 CM, or CM from MDA‐MB‐231 pretreated with 15 μM GW405833 (C) 15 μM GW405833 alone, or MDA‐MB‐231 CM in the presence or absence of 15 μM GW405833 on UMR‐106 cells and (D) comparative and combined exposure routes of GW405833 on UMR‐106 cell viability under MDA‐MB‐231‐derived CM exposure: Cells exposed to MDA‐MB‐231 CM from 15 μM GW405833 pretreated cells (Pre), cells exposed to MDA‐MB‐231 CM co‐treated with 15 μM GW405833 (Co), or the combination of both conditions (Pre + Co). The results were expressed as mean ± SEM of three independent biological replicates with three technical replicates each. (*p < 0.05, **p < 0.01, ***p < 0.001, ****p < 0.0001, while #*p* < 0.05, ##*p* < 0.01, ####*p* < 0.0001).

To investigate the response of cells to GW405833, MDA‐MB‐231 breast cancer cells and UMR106 osteoblast cells were tested for their sensitivity to the compound. We found that MDA‐MB‐231 is highly responsive to GW405833 by showing a significant reduction in cell viability (Figure 1A). Nevertheless, UMR‐106 showed a less cytotoxic effect for GW405833 (Figure 1B). The IC_50_ of GW405833 in MDA‐MB‐231 was 16.46 μM, while it was 94.01 μM in UMR‐106 cells, which was a 5.71‐fold difference (Figure 1C). Besides, our previous study showed the negative effect of breast cancer (BCa) MDA‐MB‐231 cell‐derived CM (MDA‐MB‐231 CM) on osteoblast UMR‐106 cell viability, while pretreatment with GW405833 on breast cancer cells effectively mitigated this effect [12]. The phenomenon was confirmed again in this study as shown in Figure 2B where MDA‐MB‐231 CM reduced osteoblast viability by 33%, and the pre‐exposure of GW405833 on BCa cells recovered osteoblast viability significantly (Figure 2B). We further investigated the direct effects of GW405833 on osteoblast UMR‐106 cells and if the protective effects of GW405833 on osteoblast viability could also be seen when the treatment was applied directly on osteoblasts. Our results showed that GW405833 notably increased UMR‐106 cell viability (Figure 2C) To evaluate the direct effect of CB2 agonists on UMR‐106 cell viability under MDA‐MB‐231 CM exposure, UMR‐106 cells were treated with MDA‐MB‐231 CM together with GW405833. As shown in Figure 2C, while MDA‐MB‐231 CM drastically suppressed UMR‐106 cell viability, osteoblast cell viability was significantly recovered by 23% in the presence of GW405833 as compared to osteoblasts exposed to MDA‐MB‐231 CM alone (Figure 2C). As the exposure of GW405833 on both BCa and osteoblast cells could significantly recover the deleterious effects of breast cancer‐derived CM on osteoblast cell survival, an additional experiment was carried out to compare and evaluate the effects of GW405833 exposure on BCa before CM collection (pretreatment; pre), on osteoblasts together with the exposure of BCa CM (cotreatment; co) and the combination of both methods (pretreatment + cotreatment; pre + co). Our findings indicated that pretreatment of GW405833 on MDA‐MB‐231 exhibited a more pronounced recovery effect on UMR‐106 viability under BCa CM exposure as compared to the cotreatment group. Interestingly, the combined pretreatment and cotreatment condition did not significantly differ from the individual exposure of GW405833 on each side, indicating that the synergistic effect from the two exposure routes was not seen (Figure 2D). These results indicated that GW405833 exposure could interfere with BCa‐induced osteoblast cell death on both sides of this interaction.

### 3.2 GW405833 Reduced p‐ERK and AKT Phosphorylation but Did Not Alter mTOR Protein Expression and Its Phosphorylation in MDA‐MB‐231 Cells

**FIGURE 3:**
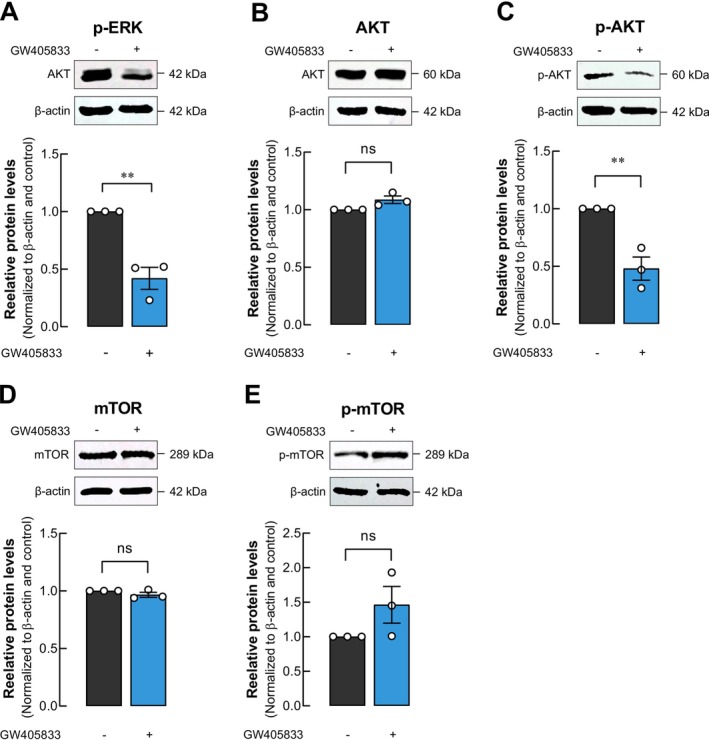
p‐AKT and p‐ERK pathways were modulated in MDA‐MB‐231 cells by GW405833 exposure. Representative membrane and quantitative data of western blot displaying the levels of (A) p‐ERK, (B) total AKT, (C) phosphorylated AKT, (D) total mTOR, and (E) phosphorylated mTOR proteins normalized to β‐Actin. The results were expressed as mean ± SEM of at least three independent biological replicates each. (**p < 0.01).

To determine the affected signaling pathways that could contribute to GW405833's effect on BCa, the expression of several cell signaling proteins was examined. Since our previous report also revealed the direct negative effect of GW405833 on MDA‐MB‐231 cell proliferation, proteins associated with this mechanism in MDA‐MB‐231 cells exposed to GW405833 were determined. Our data revealed a significant reduction in the expression of phosphorylated p44/42 MAPK (Erk1/2) in MDA‐MB‐231 cells upon GW405833 exposure (Figure 3A). Moreover, the PI3K/Akt/mTOR signaling pathway plays a significant role in breast cancer progression [29, 30]; protein expression and phosphorylation of AKT and mTOR in MDA‐MB‐231 upon GW405833 exposure were investigated. Our results showed that while the level of AKT was not changed (Figure 3B), there was a significant decrease in phosphorylated AKT level in MDA‐MB‐231 cells exposed to GW405833 (Figure 3C). On the other hand, the expression levels of mTOR (Figure 3D) and its phosphorylation (Figure 3E) showed no significant differences upon GW405833 exposure. These results suggested that GW405833 influenced ERK signaling and reduced AKT phosphorylation without affecting mTOR protein in MDA‐MB‐231 cells.

### 3.3 GW405833 Induced AKT/mTOR Protein Expression and Phosphorylation in UMR‐106 Cells

**FIGURE 4:**
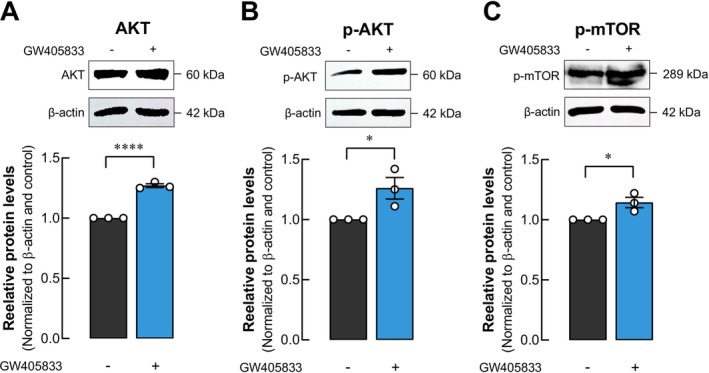
GW405833 Regulated AKT, p‐AKT, and p‐mTOR pathways in UMR‐106 cells. Representative membrane and quantitative data of western blot displaying the levels of (A) total AKT, (B) phosphorylated AKT, and (C) phosphorylated mTOR proteins normalized to β‐Actin. The results were expressed as mean ± SEM of three independent biological replicates each. (*p < 0.05, ****p < 0.0001).

Given the observed growth promotion of UMR‐106 cells upon GW405833 exposure as well as the direct protective effects of GW405833 on UMR‐106 cell viability under MDA‐MB‐231 CM treatment (Figure 2), the levels of proteins in AKT/mTOR were elucidated in UMR‐106 cells exposed to GW405833. Our results revealed a significant induction in both AKT and its phosphorylated form following the exposure to GW405833 (Figure 4A,B). Interestingly, the phosphorylated form of mTOR was notably increased (Figure 4C). These findings indicated that the AKT/mTOR pathway was associated with the positive effects of GW405833 in osteoblasts.

### 3.4 mTOR Inhibitor Reversed the Recovery Effect of GW405833 on UMR‐106 Cell Viability Under MDA‐MB‐231‐Derived CM Exposure

**FIGURE 5:**
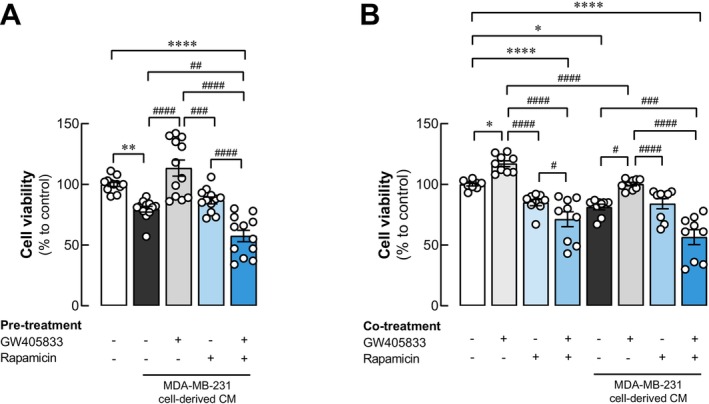
The inhibition of mTOR upon rapamycin treatment revealed the involvement of mTOR in a negative effect of MDA‐MB‐231 derived conditioned media on UMR‐106 cells. Cell viability was determined by MTT assay. UMR‐106 cells were treated with (A) MDA‐MB‐231 CM from control MDA‐MB‐231 cells and MDA‐MB‐231 cells pretreated with 15 μM GW405833, 30 nM rapamycin, and the combination of both reagents, (B) UMR‐106 cells were exposed to MDA‐MB‐231 in the presence of 15 μM GW405833, 5 nM rapamycin, and the combination of both. The results were expressed as mean ± SEM of at least three independent biological replicates with three technical replicates each. (*p < 0.05, **p < 0.01, ****p < 0.0001, while #*p* < 0.05, ##*p* < 0.01, ###*p* < 0.001, ####*p* < 0.0001).

Our results in Figure 4 showed that the AKT/mTOR pathway may associate with GW405833 effects on osteoblast survival. Since mTOR lies downstream of AKT activation [31], in this study, rapamycin, an mTOR inhibitor, was used to determine the effects of AKT/mTOR inhibition on the recovery effects of GW405833 on UMR‐106 cells under the indirect interaction with MDA‐MB‐231 CM. Firstly, UMR‐106 cells were exposed to CM collected from MDA‐MB‐231 cells pretreated with GW405833, rapamycin, or the combination of these chemicals before CM collection. Our results confirmed the recovery effects of GW405833 on MDA‐MB‐231 CM‐induced osteoblast cell growth suppression, as shown in Figures 2B and 5A. The exposure of rapamycin on MDA‐MB‐231 alone did not affect the capability of MDA‐MB‐231 CM to suppress UMR‐106 cell viability. Interestingly, when CM was collected from MDA‐MB‐231 cells pre‐exposed to both GW405833 and rapamycin, the recovery effects of GW405833 were canceled (Figure 5A). Further, the cotreatment experiments were also performed by exposing GW405833, rapamycin, and their combination on UMR‐106 cells in the presence or absence of MDA‐MB‐231 CM. Our results showed that GW405833 increased UMR‐106 cell viability, as rapamycin had no significant effect. The combination of both agents; however, negatively impacted UMR‐106 cell growth (Figure 5B). Under the presence of MDA‐MB‐231 CM, our data confirmed that MDA‐MB‐231 CM reduced UMR‐106 cell survival, and cotreatment of GW405833 recovered this effect (Figures 2C,D and 5B). On the other hand, rapamycin canceled the recovery effect of GW405833 on UMR‐106 cell viability under MDA‐MB‐231 CM exposure (Figure 5B). Our results indicated the involvement of the AKT/mTOR pathway in the recovery effects of GW405833 on UMR‐106 cell viability.

### 3.5 GW405833 Suppressed Osteoblast‐Enhanced Breast Cancer Cell Invasion and the Expression of Cancer Invasion‐Related Proteins in Both MDA‐MB‐231 and UMR‐106 Cells

**FIGURE 6:**
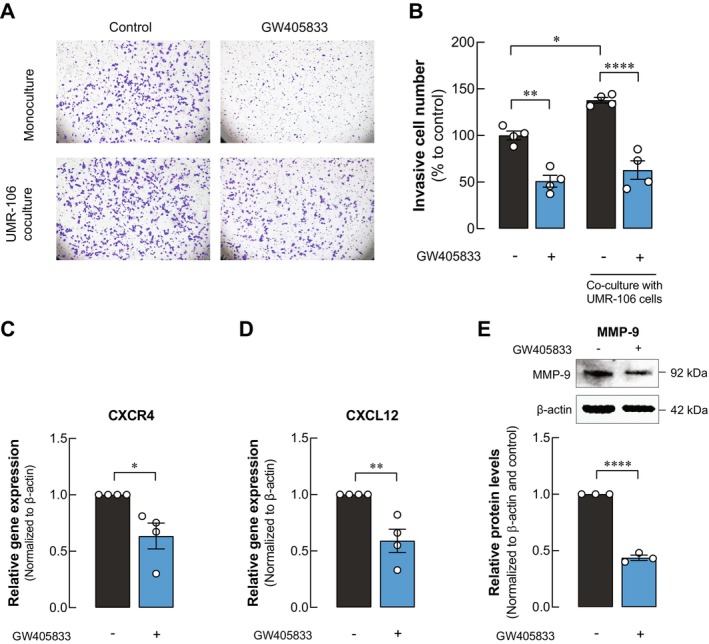
GW405833 decreased MDA‐MB‐231 invasion in the UMR‐106 co‐culture system through CXCR4/CXCL12/MMP9 axis. (A) Representative images of invasive MDA‐MB‐231 cells cultured with or without UMR‐106 cells and treated with or without GW405833, assessed by transwell invasion assay. (B) Quantitative data showed the percentage of invasive cells as compared to control. (C) The protein expression of CXCR4 in MDA‐MB‐231 exposed to GW405833. (D) The expression of CXCL12 in UMR‐106 cells exposed to GW405833. (E) Representing western blot analysis and its quantitative data for MMP‐9 level in MDA‐MB‐231 cells exposed to GW405833. Each experiment represents mean ± SEM from at least three independent biological replicates. (*p < 0.05, **p < 0.01, ****p < 0.0001).

Given that invasion is a key characteristic in breast cancer metastasis, we co‐cultured the invasive breast cancer cell line MDA‐MB‐231 with the osteoblast cell line UMR‐106 to examine the impact of GW405833 on the invasive behavior of MDA‐MB‐231 in the presence of osteoblasts. Interestingly, the results demonstrated that MDA‐MB‐231 invasion was notably enhanced when co‐cultured with UMR‐106. However, GW405833 significantly suppressed MDA‐MB‐231 invasion both in monoculture and in the presence of osteoblasts in co‐culture conditions (Figure 6A,B). Since chemokine receptor C‐X‐C chemokine receptor type 4 (CXCR4) on MDA‐MB‐231 and its ligand C‐X‐C motif chemokine ligand 12 (CXCL12) from bone cells play a crucial role in MDA‐MB‐231 bone metastasis [32], we explored the impact of GW405833 exposure on the expression of these two genes in breast cancer and osteoblasts, respectively. The results revealed a significant reduction in CXCR4 expression in MDA‐MB‐231 cells (Figure 6C) and CXCL12 expression in UMR‐106 cells (Figure 6D) following GW405833 exposure. Furthermore, the expression of MMP‐9, an enzyme functioning in cancer invasion [33] was also investigated. Our results showed a significant decrease in MMP‐9 protein levels upon GW405833 exposure as compared to the control (Figure 6E). All these changes corresponded to the reduced MDA‐MB‐231 invasion in the presence of GW405833.

### 3.6 GW405833 Reduced the Expression of Osteoclastogenic Factors Induced by MDA‐MB‐231 CM in UMR‐106 Cells

**FIGURE 7:**
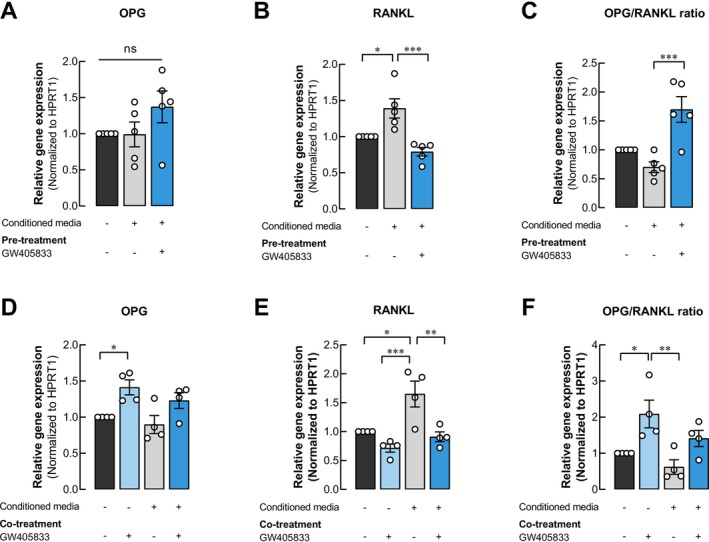
GW405833 counteracted the effect of MDA‐MB‐231‐derived conditioned media by modulating the OPG/RANKL expression ratio. The relative expression normalized to HPRT1 of (A, D) OPG, (B, E) RANKL, (C, F) OPG/RANKL ratio on UMR‐106 cells treated with (A–C) CM from MDA‐MB‐231 cells or the cells pretreated with GW405833 and (D–F) MDA‐MB‐231 CM together with or without GW405833. Each experiment was represented by the mean ± SEM of at least four independent biological replicates. (*p < 0.05, **p < 0.01, ***p < 0.001).

Osteoblasts regulate osteoclast differentiation through the secretion of nuclear factor (NF)‐κB ligand (RANKL) and osteoprotegerin (OPG). In our study, we investigated the impact of GW405833 on OPG/RANKL expression in UMR‐106 cells treated with breast cancer cell‐derived conditioned media (CM). Our results showed that RANKL expression was significantly increased in UMR‐106 cells exposed to MDA‐MB‐231 CM, while OPG expression was unaffected (Figure 7A,B). The altered expression resulted in the slight decrease in OPG/RANKL expression ratio in UMR‐106 cells exposed to MDA‐MB‐231 CM (Figure 7C). In contrast, when CM was collected from MDA‐MB‐231 pre‐exposed to GW405833, this could reverse the stimulatory effect of MDA‐MB‐231 CM on RANKL expression in UMR‐106 cells (Figure 7B), and the OPG/RANKL expression ratio in UMR‐106 cells was further enhanced after the exposure of CM from GW405833 pre‐exposed MDA‐MB‐231 cells (Figure 7C). Furthermore, this phenomenon was also examined under the conditions when UMR‐106 cells were simultaneously exposed to MDA‐MB‐231 and GW405833. Our results showed that GW405833 enhanced OPG gene expression in UMR‐106 cells (Figure 7D). While the enhancing effect of MDA‐MB‐231 CM on RANKL expression in UMR‐106 cells was confirmed again in Figure 7E, this induction was suppressed in the presence of GW405833 (Figure 7E). With this change, the expression ratio between OPG and RANKL was upregulated under GW405833 exposure. Even though the result was not statistically significant, OPG/RANKL showed an increasing trend in the presence of GW405833 even under the exposure of MDA‐MB‐231 CM (Figure 7F). These results suggested the regulatory potential of GW405833 in breast cancer‐osteoblast interaction in the OPG/RANKL axis.

### 3.7 MDA‐MB‐231 CM Enhanced Osteoclastogenesis, While GW405833 Exposure Suppressed This Effect

**FIGURE 8:**
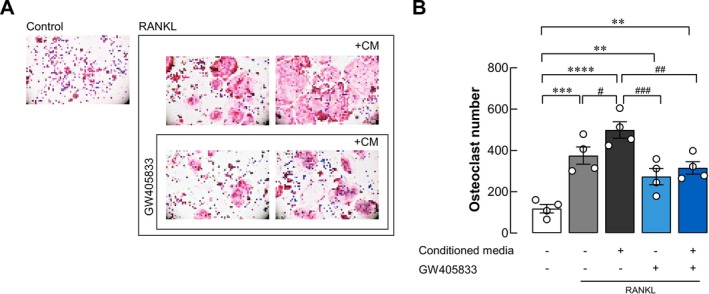
GW405833 suppressed osteoclastogenic effects from MDA‐MB‐231‐derived conditioned media. (A) Representative images of TRAP staining in RANKL‐primed RAW264.7 cells treated with 10% MDA‐MB‐231‐derived CM, GW405833, or both, with and without RANKL exposure (B) quantifies the number of osteoclasts containing at least three nuclei from the experiments depicted in (A). The results were expressed as mean ± SEM of four independent biological replicates. (**p < 0.01, ***p < 0.001, ****p < 0.0001, #*p* < 0.05, ##*p* < 0.01, ###*p* < 0.001).

Breast cancer bone metastasis promotes bone degradation [34], primarily mediated by osteoclasts. This study aimed to investigate the impact of MDA‐MB‐231 cell‐derived CM on osteoclast differentiation, as well as the effects of GW405833 exposure on this interaction. Osteoclast differentiation was assessed using a tartrate‐resistant acid phosphatase (TRAP) staining assay with RAW264.7 cells as osteoclast progenitors. When RAW264.7 cells were treated with MDA‐MB‐231 cell‐derived CM, a significant enhancement in osteoclastogenesis was observed. In contrast, exposure to GW405833 suppressed osteoclast differentiation. Additionally, the presence of RANKL further enhanced the osteoclastogenic effect of MDA‐MB‐231 CM, which was markedly reduced by GW405833 (Figure 8A,B).

## 4 Discussion

The intricate interplay between breast cancer and bone cells plays a pivotal role in cancer bone metastasis. Breast cancer cells exert regulatory control over bone cell differentiation and activity to facilitate their colonization in the bone microenvironment by disrupting bone formation and causing osteolytic lesions [34]. Previous studies have reported the induction of osteoblast cell death by conditioned media (CM) collected from breast cancer cells [12, 35]. Consistent with this study, our investigation confirmed that CM derived from breast cancer MDA‐MB‐231 cells inhibited the viability of osteoblastic UMR‐106 cells. Osteoblasts can be regulated by the endocannabinoid system (ECS), particularly through the cannabinoid receptor 2 (CB2). The activation of CB2 has been shown to promote bone formation by influencing osteoblasts and their precursors [17]. On the other hand, its absence resulted in a lack of osteogenic induction as observed in CB2‐deficient mice [36]. Furthermore, studies have demonstrated the expression of CB2 receptors and their ability to enhance osteoblast activity and functions [37]. Our findings align with the beneficial role of CB2 in maintaining bone metabolism and promoting osteoblast cell viability. Specifically, the CB2 agonist (GW405833) demonstrated a positive impact on the viability of UMR‐106 cells. Beyond its positive effects on bone formation, our data highlighted an additional aspect of how the CB2 agonist may contribute to the protection of the bone microenvironment during breast cancer bone metastasis. In our study, we observed GW405833 was able to restore UMR‐106 cell viability under exposure to breast cancer CM. This suggested a potential protective effect of the CB2 agonist against the detrimental impact of breast cancer‐derived factors on osteoblast cells. This result corresponded with the inhibitory effect of the CB2 agonist on breast cancer bone colonization and cancer‐induced bone loss in animal studies [38, 39]. Interestingly, our results also demonstrated that GW405833 could disrupt breast cancer CM‐mediated osteoblast suppression both on the breast cancer side and the osteoblast side. Accordingly, GW405833 may inhibit the secretion of factors contributing to osteoblast damage in breast cancer cells. At the same time, its direct positive impact on osteoblast survival was also observed even under the presence of breast cancer CM.

In addition to the positive effects of CB2 agonist on bone formation and its protective roles on osteoblast during osteoblast‐breast cancer interaction, the anticancer effects of CB2 agonists were also reported in many cancers [23, 40, 41, 42]. Similarly, our previous report also showed that GW405833 significantly suppressed breast cancer cell survival in MDA‐MB‐231 cells [12]. With its breast cancer growth inhibition, the expression of proteins involved in cell proliferation was investigated in MDA‐MB‐231 exposed to GW405833 in this study. Our results showed that GW405833 significantly suppressed ERK phosphorylation in MDA‐MB‐231 cells. These results correlated with reduced breast cancer cell survival reported in our previous study [12]. Several studies also reported the association between ERK phosphorylation and breast cancer progression [43, 44]; hence, our results suggested the potential anticancer mechanism of GW405833 in MDA‐MB‐231 to be associated with ERK phosphorylation suppression. Another key signaling pathway mediated by CB2 activation is the PI3K/AKT/mTOR pathway [29, 30]. The signaling cascade was shown to control cell quiescence, cell cycle, and cell growth in breast cancer [45]. A previous study from our group revealed that GW405833 reduced the phosphorylation of NF‐κB, a downstream molecular component of the PI3K/AKT/mTOR pathway corresponding to its anticancer activity in MDA‐MB‐231 cells [12]. This observation also correlated with the decreased level of phosphorylated AKT in MDA‐MB‐231 cells exposed to GW405833 reported in this study. In addition to its role in breast cancer cell growth, the PI3K/AKT/mTOR pathway was shown to regulate breast cancer bone metastasis [46]. Even though the result is not statistically significant, the increased trend of phosphorylated mTOR was noted in this study, and the reciprocal correlation between phosphorylated AKT and mTORC1 was also reported in cancer [47]. Accordingly, the involvement of the PI3K/AKT/mTOR pathway in the recovery effects of GW405833 in MDA‐MB‐231 CM‐induced osteoblast cell death was investigated. With its aforementioned roles in breast cancer development, the PI3K/AKT/mTOR pathway was shown to promote osteoblast survival, differentiation, and function and to facilitate the synthesis and deposition of new bone matrix during bone formation [31, 48]. In contrast to the effects seen in MDA‐MB‐231 cells, our study revealed a notable induction of AKT and mTOR phosphorylation in UMR‐106 cells following exposure to GW405833. Our results from previous and current studies indicated that while GW405833 suppressed breast cancer MDA‐MB‐231 survival and interfered with its interaction with osteoblasts, GW405833 supported osteoblast cell survival, potentially through the differential effects of GW405833 on the PI3K/AKT/mTOR pathway in these cells. Interestingly, our study revealed that an mTOR inhibitor (rapamycin) reversed the protective effect of GW405833 on UMR‐106 cells exposed to MDA‐MB‐231 CM. On the other hand, rapamycin could also reverse the stimulatory effect of GW405833 on UMR‐106 cell viability, both in the presence and absence of MDA‐MB‐231‐derived CM. These results suggested that the AKT/mTOR pathway also played a crucial role in the positive impact of GW405833 on osteoblast. Accordingly, GW405833 may interfere with breast cancer and osteoblast interaction by manipulating the AKT/mTOR pathway both in breast cancer and in osteoblast.

Metastatic breast cancer cells possess the ability to migrate on extracellular matrix (ECM) as well as to invade through ECM during the metastatic cascade. Our previous study found that GW405833 could inhibit breast cancer cell migration [12]. For breast cancer bone metastasis, the migration of breast cancer cells is guided by chemoattractants from bone [49]. Interestingly, our results demonstrated an enhancement of MDA‐MB‐231 cell invasion when co‐cultured with osteoblast UMR‐106 cells. However, GW405833 could suppress MDA‐MB‐231 invasion in both the presence and absence of osteoblasts. The chemokine receptor CXCR4 and its ligand CXCL12 play a significant role in breast cancer cell colonization in bone [32]. Breast cancer cells that exhibit an elevated expression of CXCR4 have a greater tendency to be attracted by CXCL12, which is secreted by osteoblasts and plays a role in attracting osteogenic precursors to bone marrow [50, 51]. Our results revealed a significant reduction in CXCR4 expression in MDA‐MB‐231 cells and CXCL12 expression in UMR‐106 cells following GW405833 treatment. Furthermore, breast cancer cells also produced matrix metalloproteinases (MMPs) to facilitate cancer cell invasion through ECM. MMP‐9 has been shown to correlate with poor survival and a short recurrence‐free period of breast cancer patients [52]. Suppression of MMP‐9 has been shown to reduce the migration and invasion ability of breast cancer cells [53]. Interestingly, our results observed a significant reduction in MMP‐9 protein level in breast cancer cells exposed to GW405833. Our results suggested potential anti‐metastatic mechanisms of GW405833 in breast cancer by modulating key molecular players involved in breast cancer cell invasion and its interaction with the bone microenvironment.

Breast cancer was shown to predominantly cause osteolytic or bone‐degrading lesions [34]. As our results showed the negative impact of MDA‐MB‐231 CM on osteoblast UMR‐106 cell survival, an additional experiment on bone‐degrading cells (osteoclasts) also revealed the activation of osteoclast differentiation upon the exposure to MDA‐MB‐231 CM. These results corresponded to previous reports showing that factors from breast cancer cells could facilitate osteoclast development and bone degradation during breast cancer bone metastasis [54, 55, 56]. The presence of GW405833; however, suppressed this interaction and reduced the effects of MDA‐MB‐231 CM on osteoclast precursor cells. The inhibitory role of CB2 activation in osteoclast differentiation was also reported in iron‐induced and RANKL‐induced osteoclastogenesis [57, 58]. However, in the context of bone remodeling, osteoblasts also engage in dynamic interactions with osteoclasts. This interplay involves RANKL, produced by osteoblasts to promote osteoclast differentiation, and OPG, acting as a decoy receptor for RANKL to inhibit bone resorption and regulate bone homeostasis [59]. Breast cancer‐derived cytokines contributed to a decreased OPG:RANKL ratio in osteoblasts and bone stromal cells [56, 60, 61]. In addition, a lower OPG:RANKL ratio was shown to correlate with breast cancer bone metastasis. Our findings aligned with these observations as breast cancer CM promoted the expression of RANKL in osteoblasts. Furthermore, GW405833 exposure on breast cancer (pretreatment) suppressed CM‐induced RANKL expression, leading to an increased OPG/RANKL ratio in osteoblasts. Our data indicated the potential of GW405833 to mitigate osteoclast differentiation induced by MDA‐MB‐231 CM, which could reduce the risk of osteolytic lesions via the modulation of RANKL/OPG pathways. In conclusion, our data indicated the potential protective effects of GW405833 on breast cancer‐mediated osteolysis by at least two processes. Firstly, the presence of GW405833 interfered with the deleterious effects of breast cancer on osteoblasts through the modulation of both breast cancer cells and osteoblasts. Secondly, GW405833 suppressed breast cancer CM‐mediated osteoclast differentiation and increased the OPG:RANKL ratio in osteoblasts. As the underlying mechanisms of these phenomena are still unclear, our study revealed that the AKT/mTOR pathway is involved in breast cancer and osteoblast interaction. While further study is still needed, our results broaden the understanding and therapeutic targets for breast cancer bone metastasis.

## Author Contributions

**Ingon Inson:** data curation, formal analysis, investigation, methodology, validation, visualization, writing – original draft, review, and editing. **Chartinun Chutoe:** investigation, methodology, software, visualization, writing – original draft, review, and editing. **Janjira Kanjanapipak:** data curation, investigation, methodology, validation. **Kornkamon Lertsuwan:** conceptualization, formal analysis, validation, funding acquisition, methodology, project administration, resources, supervision, writing – original draft, review, and editing.

## Conflicts of Interest

The authors declare no conflicts of interest.

## Supporting information


Figure S1.
Figure S2.
Figure S3.
Table S1.

## Data Availability

The data that support the findings of this study are available from the corresponding author upon reasonable request.
