# Supplementary material for: Cannabinoid Receptor Type 2 Agonist, GW405833, Reduced the Impacts of MDA‐MB‐231 Breast Cancer Cells on Bone Cells
Source: Cancer Med. 2025 Feb 20;14(4):e70709. doi: 10.1002/cam4.70709 (PMC11842928; doi:10.1002/cam4.70709)
Supplement: Supplementary file 1 — Figure S1. Figure S2. Figure S3. Table S1. [file CAM4-14-e70709-s001.docx]

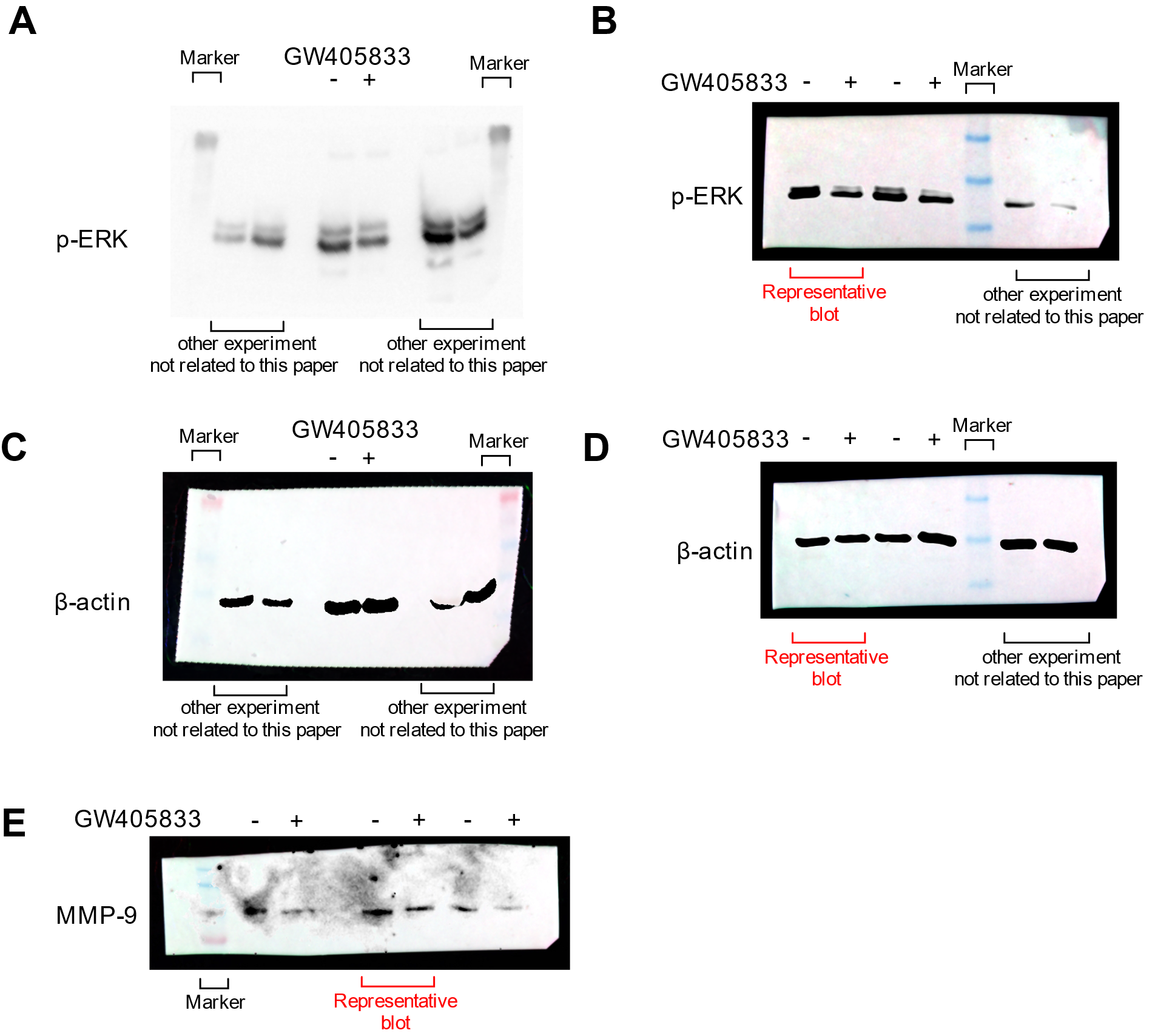
­

**Supplementary Figure 1.** Original Western blot for three biological replicates corresponding to Figure 3 and 6. **(A)** Whole blot representing p-ERK (44 and 42 kDa) from biological replicate 1 after membrane cutting. **(B)** Whole blot representing p-ERK (44 and 42 kDa) from biological replicates 2 and 3 after membrane cutting. **(C)** Whole blot representing β-actin (42 kDa) from biological replicate 1 after stripping and reprobing membrane A. **(D)** Whole blot representing β-actin (42 kDa) from biological replicates 2 and 3 after stripping and reprobing membranes B and biological replicates 1 and 2 of membrane B. **(E)** Whole blot representing MMP-9 (92 kDa) of three biological replicates after membrane cutting, which were normalized to their own β-actin as shown in supplementary figure 2C.


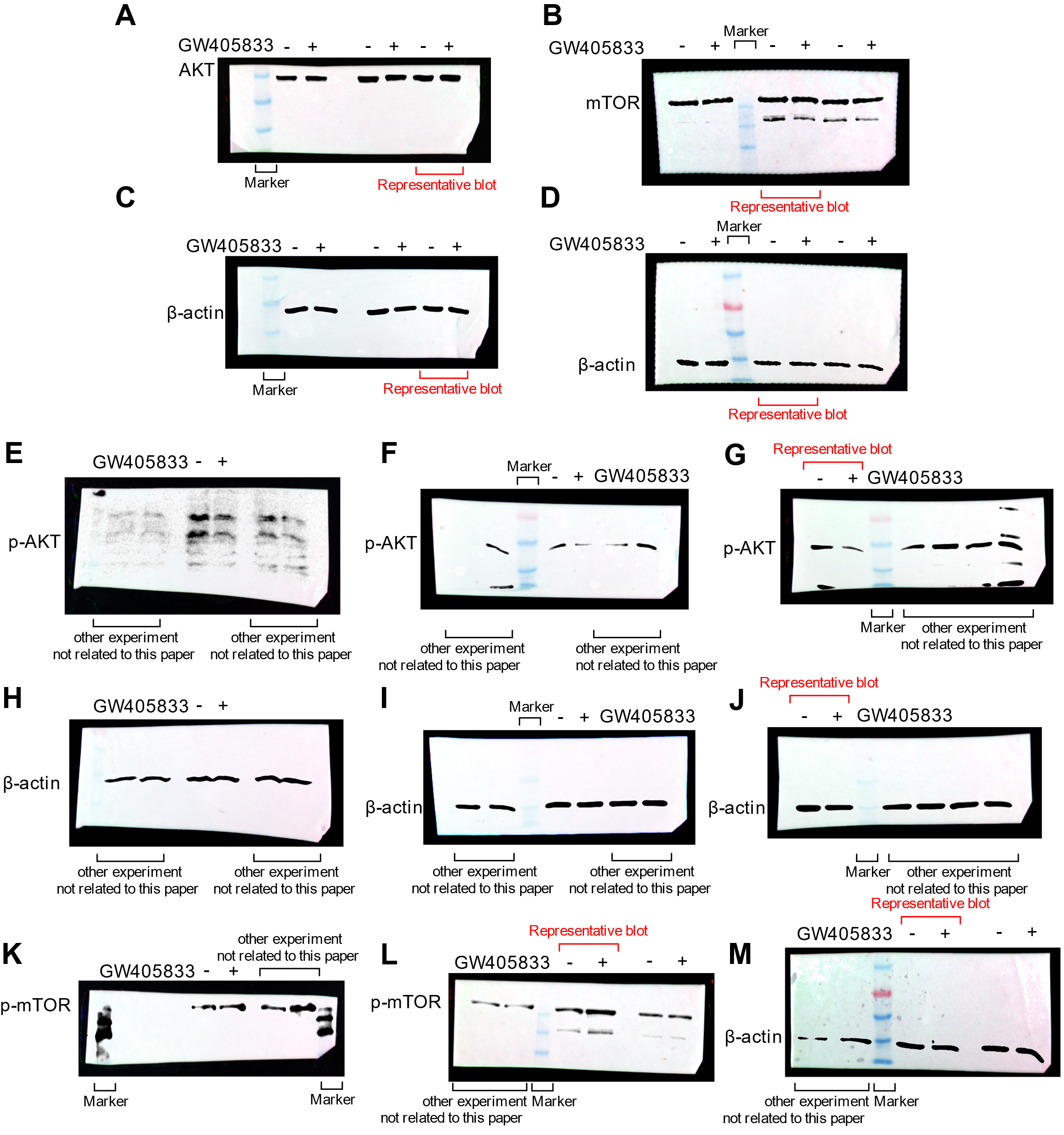


**­­­­­Supplementary Figure 2.** Original Western blot for three biological replicates corresponding to Figure 3. **(A)** Whole blot representing AKT (60 kDa) from three biological replicates after membrane cutting. **(B)** Whole blot representing mTOR (289 kDa) from three biological replicates after membrane cutting. **(C)** Whole blot representing β-actin (42 kDa) from three biological replicates after stripping and reprobing membrane A. **(D)** Whole blot representing β-actin (42 kDa) from three biological replicates after membrane cutting, separate from membrane B. **(E)** Whole blot representing p-AKT (60 kDa) from biological replicate 1 after membrane cutting. **(F)** Whole blot representing p-AKT (60 kDa) from biological replicate 2 after membrane cutting. **(G)** Whole blot representing p-AKT (60 kDa) from biological replicate 3 after membrane cutting. **(H)** Whole blot representing β-actin (42 kDa) from biological replicate 1 after stripping and reprobing membrane E. **(I)** Whole blot representing β-actin (42 kDa) from biological replicate 2 after stripping and reprobing membrane F. **(J)** Whole blot representing β-actin (42 kDa) from biological replicate 3 after stripping and reprobing membrane G. **(K)** Whole blot representing p-mTOR (289 kDa) from biological replicate 1 after membrane cutting. **(L)** Whole blot representing p-mTOR (289 kDa) from biological replicates 2 and 3 after membrane cutting. **(M)** Whole blot representing β-actin (42 kDa) from biological replicates 2 and 3 after membrane cutting, separate from membrane L.


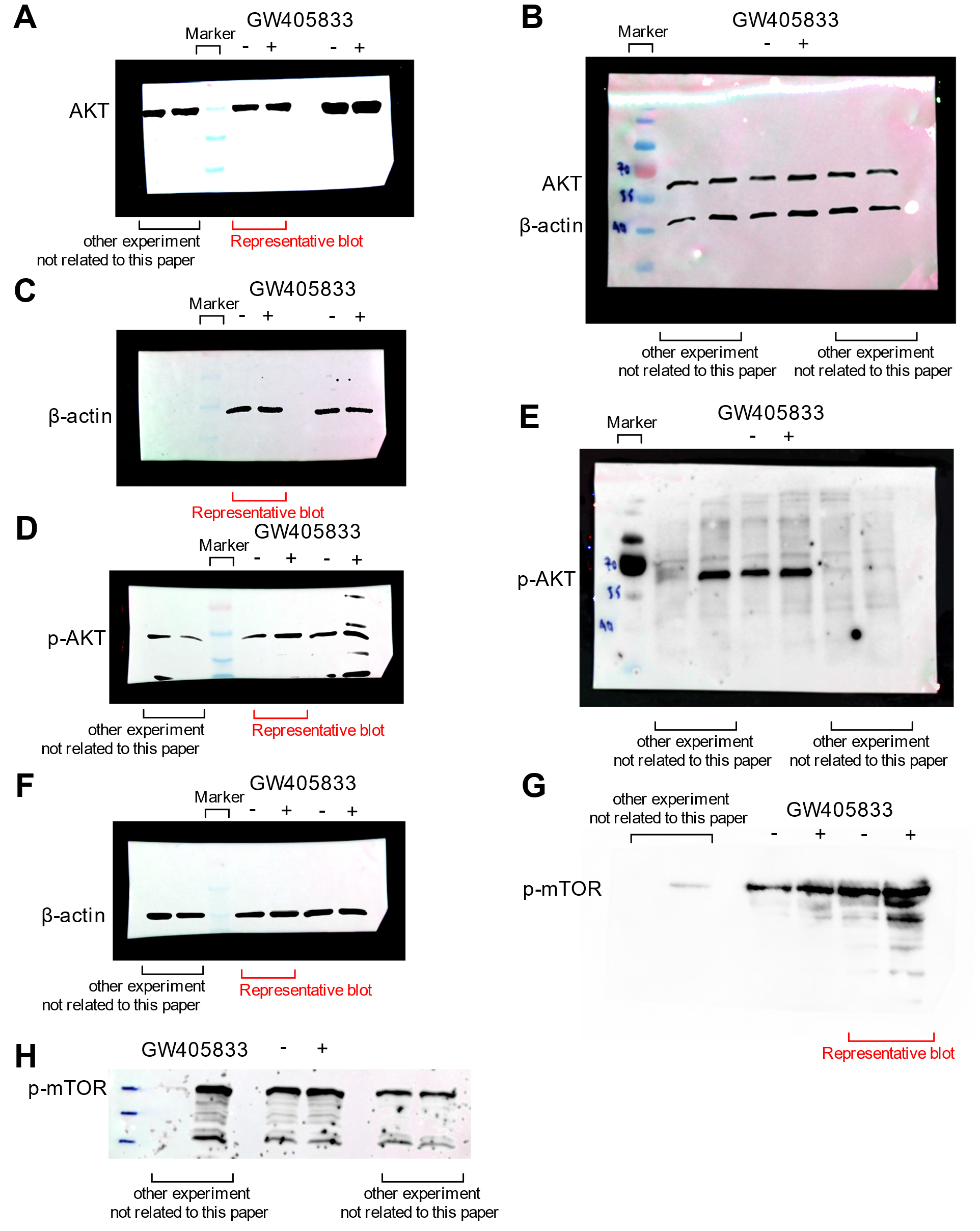


**Supplementary Figure 3.** Original Western blot for three biological replicates corresponding to Figure 4. **(A)** Whole blot representing AKT (60 kDa) from biological replicates 1 and 2 after membrane cutting. **(B)** Whole blot representing AKT (60 kDa) and β-actin (42 kDa) from biological replicate 3 after stripping and reprobing membrane E. **(C)** Whole blot representing β-actin (42 kDa) from biological replicates 1 and 2 after stripping and reprobing membrane A. **(D)** Whole blot representing p-AKT (60 kDa) from biological replicates 1 and 2 after membrane cutting. **(E)** Whole blot representing p-AKT (60 kDa) from biological replicate 3 after membrane cutting. **(F)** Whole blot representing β-actin (42 kDa) from biological replicates 1 and 2 after stripping and reprobing membrane D. **(G)** Whole blot representing p-mTOR (289 kDa) from biological replicate 1 and 2 after membrane cutting, separate from membrane F. **(H)** Whole blot representing p-mTOR (289 kDa) from biological replicates 3 after membrane cutting, separate from membrane B.

**Table S1.** The table shows the reference figures corresponding to the original blots of each protein, which were measured accordingly by ImageJ image analysis.

| **Cell lines** | **Proteins** | **Biological**  **replicates** | **Reference figure**  (Target proteins) | **Reference figure of normalized protein**  (β-actin) |
| --- | --- | --- | --- | --- |
| MDA-MB-231 | p-ERK | 1 | S1 fig. A | S1 fig. C |
|  |  | 2* | S1 fig. B | S1 fig. D |
|  |  | 3 | S1 fig. B | S1 fig. D |
|  | MMP-9 | 1 | S1 fig. E | S2 fig. C |
|  |  | 2* | S1 fig. E | S2 fig. C |
|  |  | 3 | S1 fig. E | S2 fig. C |
|  | AKT | 1 | S2 fig. A | S2 fig. C |
|  |  | 2 | S2 fig. A | S2 fig. C |
|  |  | 3* | S2 fig. A | S2 fig. C |
|  | mTOR | 1 | S2 fig. B | S2 fig. D |
|  |  | 2* | S2 fig. B | S2 fig. D |
|  |  | 3 | S2 fig. B | S2 fig. D |
|  | p-ATK | 1 | S2 fig. E | S2 fig. H |
|  |  | 2 | S2 fig. F | S2 fig. I |
|  |  | 3* | S2 fig. G | S2 fig. J |
|  | p-mTOR | 1 | S2 fig. K | S1 fig. C |
|  |  | 2* | S2 fig. L | S2 fig. M |
|  |  | 3 | S2 fig. L | S2 fig. M |
| UMR-106 | AKT | 1 | S3 fig. A | S3 fig. C |
|  |  | 2* | S3 fig. A | S3 fig. C |
|  |  | 3 | S3 fig. B | S3 fig. B |
|  | p-AKT | 1 | S3 fig. D | S3 fig. F |
|  |  | 2* | S3 fig. D | S3 fig. F |
|  |  | 3 | S3 fig. E | S3 fig. B |
|  | p-mTOR | 1* | S3 fig. H | S3 fig. F |
|  |  | 2 | S3 fig. H | S3 fig. F |
|  |  | 3 | S3 fig. G | S3 fig. B |

“ * ” This symbol represents the representative blot that were shown in the figure.
